# Radiodensitometric study for evaluation of bone mineral density around dental implants after zoledronic acid treatment in ovariectomized rats

**DOI:** 10.4317/medoral.21706

**Published:** 2017-04-08

**Authors:** Sibel Dikicier, Emre Dikicier, Umit Karacayli, Burak Erguder

**Affiliations:** 1 Department of Prostodontics, Corlu State Hospital, Tekirdag, Turkey; 2 Department of Oral and Maxillofacial Surgery, Corlu State Hospital, Tekirdag, Turkey; 3 Department of Oral and Maxillofacial Surgery, Health Science University, Faculty of Gulhane Dentistry, Ankara, Turkey; 4 Department of Oral and Maxillofacial Surgery, Yeni Yuzyil University, Faculty of Dentistry, Istanbul, Turkey

## Abstract

**Background:**

The purpose of this study was to evaluate the effects of intravenous zoledronic acid applied systemically on osseointegration of dental implants and the surrounding bone mineral density (BMD) in the ovariectomized rats.

**Material and Methods:**

36 rats were divided into three groups: control (CTRL), ovariectomy (OVX), and ovariectomy-zoledronic acid (OVX/ZOL). The rats in the CTRL group underwent sham surgery, while rats in OVX and OVX / ZOL group underwent ovariectomy. After 12 weeks, rats from OVX / ZOL were injected with 0.04 mg/kg ZOL intravenously once a week for 6 weeks. The rats from CTRL and OVX groups were injected with 0.9% NaCl. Implants were placed in the left tibia. After 8 weeks, rats were sacrificed and tibia bones were removed for radiodensitometric examination. Digital radiographs of bones’ lateral surface were taken. The BMD was measured by using radiographic analysis software.

**Results:**

Statistically significant differences were found between all groups (*p*<0.05). While highest mean BMD values were observed in the CTRL group, the lowest were in the OVX group.

**Conclusions:**

The systemic use of ZOL has increased the bone density around the implants inserted osteoporotic rat tibia.

** Key words:**Bisphosphonate, bone density, dental implants, osteoporosis.

## Introduction

Skeletal diseases such as osteoporosis that affect the bone metabolism cause alveolar bone resorption and may lead to difficulties in the application of dental implants. Osteoporosis is an osteometabolic disease that occurs in postmenopausal women due to decrease of estrogen levels (1). This disease is characterized by decrease in the bone density, strength, and regenerative capacity as well as deterioration of microstructure. As a result, osteoporosis is considered to be a relative contraindication for dental implant applications (2).

Many studies have investigated the role of osteoporosis in dental implant healing. Medications used for osteoporosis have been reported to be effective in preventing bone loss around the inserted implant (3,4). Bisphosphonates (BP) are the most commonly preferred drugs in the treatment of osteoporosis. BP is a pyrophosphate analog that contains phosphate-carbon-phosphate bond and remains stable even when exposed to chemical and enzymatic hydrolysis. It reduces the fracture risk by increasing bone mineral density, reducing bone turnover and preventing bone resorption of osteoclasts (5). The mechanism of action of this drug is by inhibition of osteoclastic bone resorption via formation of strong bonds between hydroxyapatite crystals and BP (6).

Oral BPs are often preferred for the treatment of osteoporosis. However, there are dangerous side effects of long-term, low therapeutic dose BP treatments. Bisphosphonate-related osteonecrosis of the jaw (BRONJ) has been reported as a side effect of BP use. In the treatment of multiple myeloma and metastatic bone disease, the dose of intravenous BP, duration of use, method of application, and the time of dental surgery should be adjusted with consideration of a risk of development of BRONJ. Prior to the implant surgery each patient receiving a BP therapy should be informed about the risks of developing BRONJ (7).

Previous studies have reported that BPs increase bone density around the implant. Giro *et al.* (2) have studied the effect of alendronate treatment for estrogen deficiency on the bone density around the implant. They reported that the bone density around the implant was significantly higher in the group that used alendronate. Viera-Negron *et al.* (8) investigated the effect of alendronate on bone density around the implant and implant osseointegration and reported that in the alendronate group, the bone density surrounding the implants was significantly higher. In another study, Yıldız *et al.* (9) studied the effects of zoledronic acid on implants osseointegration and bone density around the implant and found that zoledronic acid was associated with the increased bone density. Previous studies have shown that implants were pertinent in osteoporosis animal models treated with BP.

Zoledronic acid (ZOL) is a new generation bisphosphonate that shows a high affinity to hydroxyapatite. It accumulates for a very long time in the bone mineral structure therefore, it is the most powerful drug in the BP group. Recent animal studies have investigated the effect of short-term or single subcutaneous and intravenous ZOL administration on bone density around the implant (10-12). However, the studies that look into the effects of long-term intravenous ZOL administration on bone density around the implant are limited.

Histograms are utilized in the evaluation of bone healing by using digital radiographs. High histogram value of the area under the examination means that the radiograph in that area has higher opacity. Along the same lines, when comparing bone densities of different samples in digital radiographs, measuring the histogram values gives us an idea about the changes in the bone density in that area (13). The purpose of this study was to evaluate the effect of long-term, systemic administration of ZOL on bone density around the implant in an osteoporosis rat model by using radiodensitometric analysis.

## Material and Methods

- The Study Design 

This study was conducted with a total of 36 16-week-old female Wistar rats that were housed in ambient conditions with 12-hour periods of dark and light cycles. Animals were fed with standard feed, food and water were not limited. The study was conducted with the permission of the Gulhane Military Medical Academy Animal Ethics Committee.

Animals were randomly divided into three groups (12 animals in each group): control (CTRL), ovariectomy (OVX) and ovariectomy + ZOL (OVX / ZOL). Following a 7-day acclimatization period the rats from the OVX and OVX / ZOL groups underwent an operation for bilateral ovariectomy. Before the ovariectomy procedure, animals received a single dose of intraperitoneal injection of 0.35 mg/kg Ketamine HCl and 0.5 mg/kg Xylazine to achieve anesthesia. The CTRL group underwent sham surgery. During this sham surgery, the abdomen was opened in the same way, ovaries were dissected and their anatomical position was examined, then the abdomen was closed without removing the ovaries. Twelve weeks after the surgery, rats from the OVX / ZOL group were administered a single dose of 0.04 mg / kg ZOL from the tail vein every week for 6 weeks. Meanwhile, rats from the CTRL and OVX groups received the same dose of 0.9% NaCl through the tail vein for the same amount of time.

**Figure 1:**
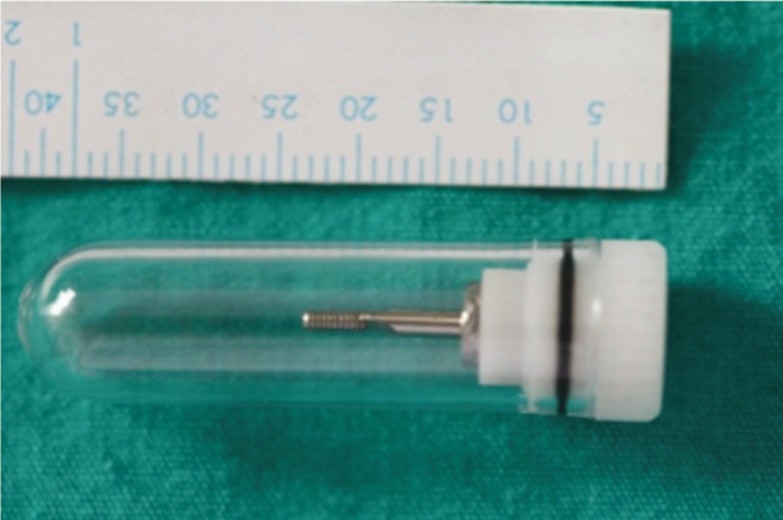
Custom made titanium micro-implant and screw.

After completion of all intravenous drug injections, animals were anesthetized by intramuscular injection of 0.35 mg / kg Ketamine HCl and 0.5 mg / kg Xylazine. Rats’ legs were shaved, washed and wiped with antiseptic iodine solution. A 20 mm incision was made on the medial of the left tibia’s proximal metaphyseal. After the procedure, the dissecting bone was removed. The bed for the bicortical implant was prepared by using a drill physiodispenser under sterile saline irrigation. The specially crafted, sandblasted titanium micro-implants with acid-etched surfaces that mimics the surface properties of the implants used in humans were utilized (Fig. 1). The micro-implants [4 mm in length and 1.6 mm in diameter] were placed in the prepared implant bed. After the implantation, fascia and skin were closed with a suture that can be resorbed in separate layers. Post-operatively the animals received a single dose of intramuscular injection of 1 mg / kg Tramadol hydrochloride and 50 mg / kg Cefazolin sodium. An 8-week bone healing period was given in order to ensure osseointegration (14). After this period, animals were sacrificed by intravenous injection of 100 mg / kg Sodium pentobarbital. Tibia bones that had implants were removed by en-bloc resection and were prepared for radiological examinations.

- Radiodensitometric analysis

The digital radiographs of tibia samples were taken with the standard test set, all from the same distance and with the same kilovolts, milliampers and exposure time (RVG, Trophy Radiologie, Vincennes, France). The sample was placed on the digital radio-graphy sensors and an X-ray was taken perpendicularly from 20 cm above the sensor’s surface (New Life Best-X DC, Torino, Italy). The X-ray parameters were adjusted to 65 KVP, 300 mA and 0.16 ms exposure time. The resolution of digital images was set to 635 ppi (pixels per inch), the size was set to 900 x 641 dpi (dots per inch) and pixel size was set to 40 µm. The resulting digital images were saved in JPEG (Joint Photographic Experts Group) format.

**Figure 2:**
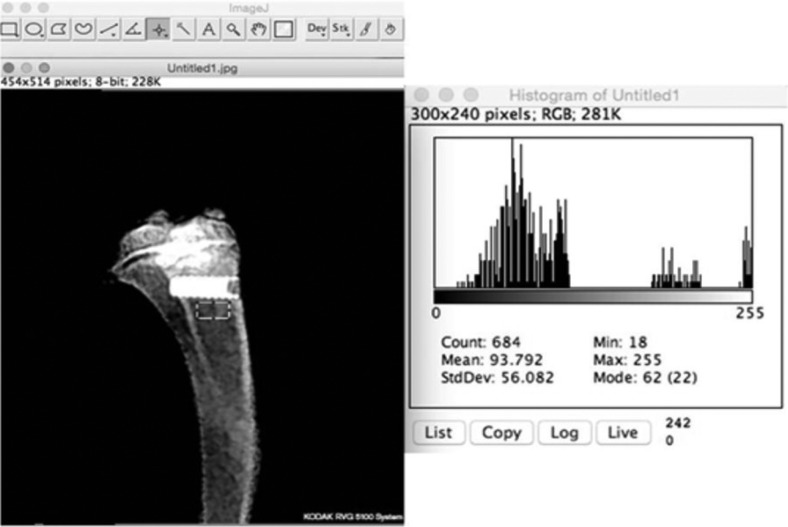
Radiological view and radiodensitometric analysis of the micro-implant.

Bone mineral density (BMD) measurements were performed by a standard computer (Intel Core i5, 2.5 GHz processor, Intel Corp., Santa Clara, CA, USA), with the OS X Yosemite (Apple, Copertino, CA, USA) operating system and 13.3 inch LED backlit flat screen (Mac Book Pro, Apple, Copertino, CA, USA) by using Image J 1.33 image analysis software (National Institutes of Health, Bethesda, MD, USA). Sampling was done from areas of equal width that were selected from the medullary bone areas that were adjacent to implant’s apical 3 grooves. The software’s histogram property, which expresses the light distribution of the digital image in one graph, was used to obtain the mean BMD values of each sample (Fig. 2).

- Statistical Analysis 

The Kolmogorov-Smirnov test was used to assess whether the variables were normally distributed. The Kruskal-Wallis test was used for comparisons between the three groups in statistical evaluation of resulting BMD values. The pair-wise comparison between the groups was made by Bonferroni corrected Mann-Whitney U test. *p* <0.05 was considered statistically significant.

## Results

**Figure 3:**
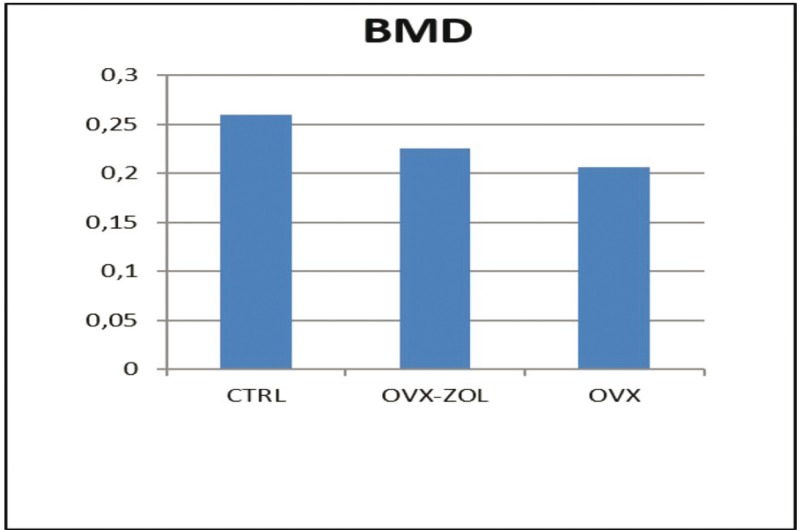
Differences in BMD parameters between CTRL, OVX/ZOL, and OVX groups. Bone mineral density (BMD), control group (CTRL), ovariectomy group(OVX), and ovariec- tomy-zoledronic acid group (OVX/ZOL).

All procedures were well tolerated by animals. One sample from each group was excluded from the radiodensitometric analysis. The means and standard deviation for BMD values are given in  Table 1. There were significant differences between the three groups in the comparison of BMD values (*p* <0.05). The highest mean BMD value was detected in the CTRL group with 94.20 ± 2.27, which was followed by OVX / ZOL group with 74.62 ± 2.43 and OVX group with 55.79 ± 2.33. The pair-wise comparisons showed that there were significant differences between CTRL and OVX groups (*p* <0.05) and between CTRL and OVX / ZOL groups (*p* <0.05) in terms of BMD values. These results show that BMD values decreased significantly in OVX group compared to the CTRL group. Moreover, the BMD values in the OVX / ZOL group were significantly higher compared to those of the OVX group (Fig. 3).

**Table 1:**
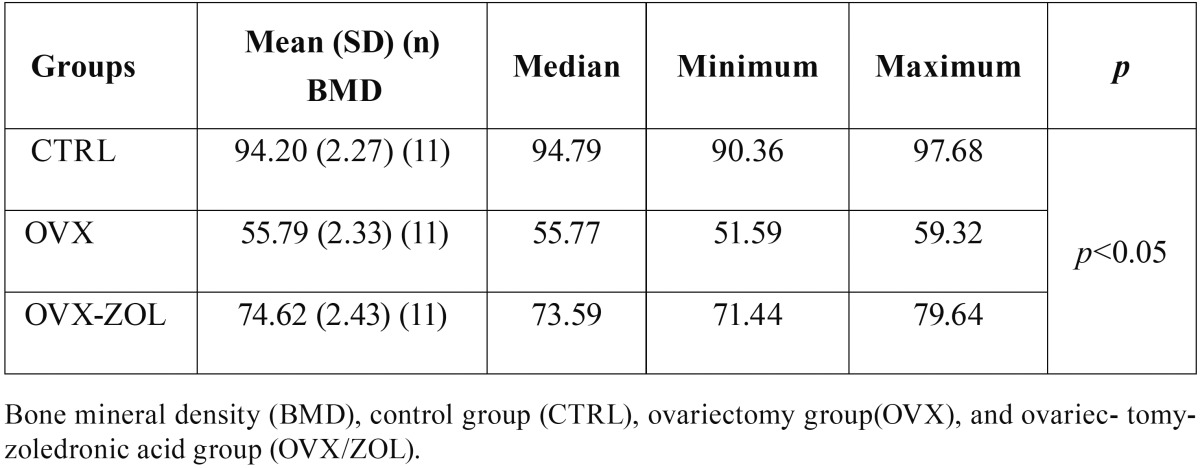
Comparison of mean BMD percentage values along with SDs of the groups as determined by radio-densitometric analysis.

## Discussion

The results of this study suggested that estrogen deficiency negatively impacts bone-implant osseointegration. The radiographic analysis of bone density showed that the bone density of the OVX group was significantly lower than those of CTRL and OVX / ZOL groups (*p*<0.05). The ovariectomy procedure applied to the OVX group and findings related to the decreased bone density are similar to studies conducted by Sakakura *et al.* (15) and Giro *et al.* (2). Moreover, administration of ZOL positively affected the bone density around the implant in the OVX / ZOL group.

Osteoporosis is a systemic skeletal disease that leads to increased bone fragility and is characterized by changes in the micro-structure and decrease in bone mass. Many studies have reported the loss of implants to be associated with the decline in patient’s medical condition. The imbalance between bone formation and degradation is observed earlier in the osteoporotic jawbone compared to long bones, as the alveolar bone is regenerated at a much faster rate. The high turnover percentage of the alveolar bone helps the mechanical durability of the maxilla and mandible, but small oral surgeries reduces local bone turnover (16). The preventive effects of osteoporosis on osseointegration of the implant, as well as causing the deficiency in the connective tissue adjacent to the implant leading to bone loss, has been reported in many studies (17-19).

The results of our study that suggested beneficial effect of intravenous BP on early bone fixation of dental implants was also highlighted in many other studies. New findings support the notion that BPs such as ibandronate, pamidronate and ZOL facilitate implant-bone fixation on healthy bone and prevent the implant loss (6).

Narai and Nagatha (4) have determined that integrated implant’s rotation torque was increased significantly in alendronate-treated ovariectomized rats compared to the healthy control group. In addition, another study has reported that osseointegration was increased in rats treated with ibandronate, however, there was no significant relationship between the degree of osseointegration and high-dose ibandronate (17).

Currently, ZOL is the most potent intravenous BP used and was approved by the Food and Drug Administration (FDA) in 2001 (20). The therapeutic use of ZOL has two preventive effects on osteoporosis: decreases bone resorption and supports bone anabolism. Furthermore, compared to the control group, ZOL has been shown to lead to two-fold increase in the bone growth around the implant (21). In this sense, in our study we benefited from systemically administered ZOL in terms of improving the osseointegration of titanium implants in ovariectomized rats.

There are various animal studies that investigated the treatment duration and dosage of ZOL. Lespessailles *et al.* (22) have determined that a single dose of intravenous 20 mg / kg ZOL lead to an increase in bone density in the ovariectomized rat model. Yıldız *et al.* (9) administered a single 0.1 mg / kg intravenous dose of ZOL to ovariectomized rabbits and 8 weeks later observed a significant improvement in bone mineralization around titanium dental implants. However, Brouwers *et al.* (23) have administered the same dose as the previous study to rats and 12 weeks later observed a significant decrease in bone density of the proximal tibia. According to the information given in this and other studies, the current protocol for intravenous administration of BP should be given in monthly doses to maintain therapeutic drug levels and to facilitate patient compliance (8). In our study, 12 weeks after the ovariectomy procedure, rats from the OVX / ZOL group were administered a single dose of 0.04 mg / kg ZOL per week for 6 weeks. This dose was chosen to simulate the cumulative dose of ZOL given to humans in clinical practice.

Many methods are used to evaluate the condition of the bone around the implant. Radiodensitometric analysis is preferred in clinical evaluation for being a rapid, non-invasive test at a low cost. In this study, the bone density image obtained from the specific area around the implant by using the histogram method was evaluated by using dedicated image analysis software. This method gave similar results in many histological studies (24,25).

In addition to inhibitory effects on osteoclasts, BP’s anabolic effects have also been highlighted in many studies. In another study, it was reported that in humans, ZOL inhibits osteoclast maturation and differentiation by directly affecting the osteoclasts (26). Therefore, these studies have linked the beneficial effects of BPs on bone density in part, to their direct effects on osteoblasts. In our study, the positive effect of ZOL on the implant osseointegration and bone structure can be explained by the anti-osteoclastic and anabolic action of ZOL.

In this study, ZOL injections began 12 weeks after the ovariectomy procedure. The implants were placed on 18th week and radiodensitometric evaluations were conducted starting from 26th week. Pan *et al.* (27) reported that after the ovariectomy, the earliest time they observed significant changes in the bone around the implant in the rat tibia was at the 12-24th week. In their radiodensitometric studies, Sakakura *et al.* (15) measured the amount of cancellous bone around the implant, which was placed in the rat’s tibia, 12 weeks after the ovariectomy procedure and determined that bone density values were significantly lower in ovariectomized rats.

Animals such as rabbits, pigs, dogs and rats are frequently used in osteoporosis studies. In our study, rat model was used because they are easy to breed, inexpensive, easier to manipulate compared to larger animals, provide the ability to obtain more samples, are resistant to infections, and because there are many resources available about their physiology.

According to our results, systemic administration of ZOL has increased the implant osseointegration in the rat model with suppressed estrogen. In addition, our radiodensitometric analyses showed that ovariectomy-related estrogen deficiency might jeopardize the bone structure. When examined in detail, the OVX group had decreased cortical bone thickness, decreased new bone formation, increased bone porosity, and limited bone-implant contact compared to other groups. However, although intravenous administration of ZOL increased the osteoporotic bone regeneration around the implant in OVX / ZOL group, the mean bone density values of this group were still lower than non-osteoporotic CTRL group.

Despite these results, implant losses can occur after dental implant surgery in some patients treated with intravenous BP. This can be explained by the increased risk of BRONJ after the surgery in patients that receive BP. As reported in many studies, oncological patients using intravenous BP face the risk for developing BRONJ after the dental procedures due to introduction of other factors such as diabetes, corticosteroid therapy, alcohol and tobacco use, and poor oral hygiene (28). It is still unclear whether the BP therapy should or should not be terminated prior to surgical procedures in order to decrease the likelihood of BRONJ development (29). However, it has been reported that BP dosages used in the treatment of osteoporosis in patients who did not undergo surgical procedures pose lower risk for developing BRONJ (30). The dental health community should understand the effects of BPs on dental implant surgeries in patients receiving intravenous BP treatment. Our study is interesting in terms of evaluating the therapeutic strategies in patients with dental implants; however, high bone turnover values in ovariectomized rats cannot be translated directly to the osseointegration process in post-menopausal human. New clinical studies are needed to better examine the long-term effects of BPs on dental implant osseointegration.

## Conclusions

Despite the limitations of this animal study, our radiodensitometric results showed that in osteoporotic rats treated with the same dosage of ZOL as used in humans, the bone densitometry values were higher compared to the group that did not receive the drug.
